# Impact of headache disorders in Italy and the public-health and policy implications: a population-based study within the Eurolight Project

**DOI:** 10.1186/s10194-015-0584-7

**Published:** 2015-12-01

**Authors:** M. Allena, T. J. Steiner, G. Sances, B. Carugno, F. Balsamo, G. Nappi, C. Andrée, C Tassorelli

**Affiliations:** Headache Science Center, C. Mondino National Neurological Institute, Via Mondino 2, 27100 Pavia (I), Italy; Department of Neuroscience, Norwegian University of Science and Technology, Edvard Griegs Gate, Trondheim, NO-7491 Norway; Division of Brain Sciences, Imperial College London, London, UK; Azienda Sanitaria Locale (ASL), Pavia, Italy; Department of Pharmaceutical Sciences, University of Basel, Basel, Switzerland; Department of Brain and Behavioural Sciences, University of Pavia, Pavia, Italy

**Keywords:** Italy, Migraine, Tension-type headache, Medication-overuse headache, Impact, Disability, Lost productivity, Health policy, Global campaign against headache

## Abstract

**Background:**

Migraine, tension-type headache (TTH) and medication-overuse headache (MOH) are disabling lifelong illnesses. The Eurolight project, a partnership activity within the Global Campaign against Headache, assessed the impact of headache disorders in ten countries in Europe using a structured questionnaire coupled with various sampling methods. Here we present the findings from the Italian population.

**Methods:**

Questionnaires were distributed to a stratified sample (*N* = 3500) of the adult (18–65 years) inhabitants of Pavia province (1.05 % of the general population), randomly selected in cooperation with the local health service. Questions included demographic and diagnostic enquries, and assessment of various aspects of impact and health-care utilisation.

**Results:**

Altogether 500 questionnaires were returned of which 487 were adequately completed for analysis (58 % female, 42 % male). Among these, gender-adjusted lifetime prevalence of headache was 82.5 %, higher in females than in males (91.2 % vs 72.4 %; *p* < 0.0001). Gender-adjusted 1-year prevalence was 74.2 % (females 87.7 %, males 61.1 %; *p* < 0.0001). The most prevalent headache type was migraine (gender-adjusted 1-year prevalence 42.9 %; females 54.6 %, males 32.5 %; *p* < 0.0001), followed by TTH (28.6 %; no gender-related difference); all causes of headache on ≥15 days/month were reported by 7.0 % of participants (females 10.6 %, males 2.0 %; *p* = 0.0002), of whom 2.1 %,, all female (*p* = 0.0064) concomitantly overused acute medications (therefore probable MOH). Only 16.6 % of responders reporting headache had received a diagnosis from a doctor, and very few (2.4 %) were taking preventative medications. Headache had negative impacts on different aspects of life: education, career and earnings, family and social life. Each person with headache had lost, on average, 2.3 days from paid work and 2.4 days from household work, and missed social occasions on 1.2 days, in the preceding 3 months. An increasing gradient for impact was observed from episodic to chronic forms of headache.

**Conclusions:**

Our study reveals that in Italy, as in other countries, migraine, TTH and MOH are highly prevalent and are associated with significant personal impact. These findings have important implications for health policy in Italy.

## Background

The World Health Organization identified headache disorders as a public health priority in 2000 [1, 2]. On a clinical level it has long been evident that primary headache disorders, migraine and tension-type headache (TTH) in particular, are often lifelong illnesses which, as well as directly causing pain and disability, hinder family and social relationships and impoverish quality of life. Epidemiologically, a large number of studies provide evidence that headache among populations is ubiquitous and common [3], although many published studies prior to the last decade focused on prevalence to the exclusion of burden. The Global Campaign against Headache, conducted by *Lifting The Burden* (LTB) in official relations with the World Health Organization [4–6], set out to fill this knowledge gap as its first priority, developing standardised methodology [7]. Population-based studies in China [8], India [9] and Russia [10] in time for inclusion in the Global Burden of Disease study 2010 (GBD2010) [11] saw migraine recognised as the seventh cause of disability worldwide, with a mean estimated global prevalence of 14.7 % among adults [11, 12]. More recently, the Global Burden of Disease Study 2013 (GBD2013), with better information still, reported migraine as the sixth highest cause of disability worldwide [13]. Importantly, GBD2013 included MOH for the first time, and it entered the top twenty causes of disability. TTH was considered relatively non-disabling but, collectively, headache disorders ranked third [14].

In Europe, meanwhile, although many studies had reported migraine prevalence, there were still knowledge gaps regarding TTH and MOH, and for all headache types there were few published reports of burden. The Eurolight Project commenced in May 2007, using modified cluster-sampling in 10 countries of Europe to assess the impact of headache [15]. Multiple scientific and lay organisations collaborated with headache experts in these countries, and not all were able to draw population-based samples. The survey used the same structured questionnaire in each country, translated into the local languages, with diagnostic questions based on ICHD-II [16] taken from the HARDSHIP questionnaire [17]. Eurolight gathered analysable data from 8271 adult participants, with participation-rates between 10.6 and 58.8 % and detectable moderate interest-bias. Gender-adjusted 1-year prevalence of any headache was 78.6 %, of migraine 35.3 %, of TTH 38.2 %, of headache on ≥15 days/month 7.2 %, of probable MOH 3.1 % [18]. Personal impact was high, with a gradient of probable MOH > migraine > TTH, and generally higher among females than males. Particularly notable were the proportions of lost useful time: 17.7 % of males and 28.0 % of females with migraine lost >10 % of days; 44.7 % of males and 53.7 % of females with probable MOH lost >20 %. The conclusions of Eurolight were: “The common headache disorders have very high personal impact in the EU, with important implications for health policy” [18].

The situation in Italy is that, Eurolight apart, few data are available from the scientific literature regarding the general population. Moreover, most published studies have limitations: they were restricted to segments of the general population (the elderly [19, 20] or adolescents [21]) or to selected types of headache (*eg*, cluster headache [22]); or they were carried out before the publication of ICHD-I [23]; or they did not assess the impact of headache. Recently, however, two surveys of the adult general population of Parma based on door-to-door interviews reported a 1-year prevalence of *definite* migraine of 24.7 % [24] and of TTH of 19.4 % [25]. The finding for migraine is in line with that of Eurolight (22.2 %) for Europe generally [18], and indicative of a major public-health concern with substantial implications for health-care policy, planning and resource-allocation. Here we present the findings of Eurolight for the Italian population in order to add to the knowledge on which that policy might be based in Italy.

## Methods

### Ethics

The National Ethics Committee of Luxembourg gave overall approval of the protocol. Further approvals in Italy were obtained from the Ethics Committee of the National Neurological Institute C. Mondino.

Data protection approval was similarly obtained centrally in Luxembourg, and data were held in compliance with national and European privacy laws.

Prospective participants received a written information sheet explaining the project and enquiry, and their purpose.

### Study design

The detailed methods of the Eurolight Project have been published elsewhere [15]. It was a cross-sectional survey of randomly-selected adults aged 18–65 years, using a structured questionnaire.

### Sampling

In Italy, the questionnaire was distributed to a representative sample of the general population living in Pavia province in Lombardy region, Northern Italy (330,000 inhabitants, as reported in 2008 by the local registry office), in collaboration with Alleanza Cefalalgici - CIRNA Foundation Onlus. Random urban (70 %) and rural (30 %) samples were drawn from this population using listings supplied by *Azienda Sanitaria Locale* (ASL) of Pavia, stratified with regard to gender (M:F ratio 1:1), age (in the range 18–65 years) and education.

The sample was 3500 individuals (1.05 % of the general population). Questionnaires and supporting documents were distributed by post to each of these, in a parcel that also contained information about the study and its objective, the informed-consent form to be signed, instructions on how to complete the questionnaire and a prepaid envelope addressed to ASL for returning the documents. No reminders were sent to those who did not respond.

### Questionnaire

The development, content and validation of the structured questionnaire have been previously described [26]. The original English version was translated into Italian following LTB’s standardised translation protocol for lay documents [27].

Demographic questions were followed by neutral screening questions for headache (“Have you ever had a headache?” and “Have you had a headache during the last year?”) and, in those screening positively, by headache-diagnostic questions based on ICHD-II and several question sets addressing impact. The last included questions enquiring into past use of the health system and medications, the HALT questionnaire [28] and two questions (How would you rate your quality of life? and How satisfied are you with your ability to perform your daily living activities?) from WHOQoL-8 [29].

### Diagnosis

Only one headache type (the most bothersome when there was more than one identified) was diagnosed in each participant. According to the algorithm used to convert responses to diagnoses, headache occurring on ≥15 days/month was first set aside from episodic headache, and diagnosed according to reported medication consumption either as probable MOH (pMOH) or other headache on ≥15 days/month. Among episodic headaches, definite migraine criteria were first applied, then those of definite TTH, which trumped probable migraine. After probable TTH, a small minority of headaches were unclassifiable. For purposes of analysis, definite and probable migraine were considered together as all-migraine, as were definite and probable TTH as all-TTH.

### Non-participants

We anticipated a low participation rate because of the means of engagement (by post). We were concerned that questionnaires were more likely to be completed and returned by those most affected by headache (interest bias). These considerations suggested our responding sample would not be representative of the initial sample. Therefore, a study of non-participants was conducted by asking (by means of advertisements in the local newspapers) those who had received the questionnaire but not returned it to complete another much shorter questionnaire published on the website of CIRNA Foundation (www.cefalea.it). The short questionnaire asked about occurrence and frequency of headache during the last year and headache features to identify probable migraine (using a migraine screening instrument [30]).

### Statistics

Categorical variables were described in terms of frequency (n) and proportions (%, with 95 % confidence intervals [CIs] where appropriate), continuous variables in terms of means and standard deviations (SDs).

Analyses were done with the SAS software (version 9.1.3, SAS Inc., Cary, NC, USA), using the Chi squared test. We considered a P value of <0.05 as statistically significant.

## Results

We received 500 questionnaires (participation rate 14.3 %), of which 487 were complete and usable for analysis (males 203 [41.7 %], females 284 [58.3 %]; mean age 43.4 ± 12.6 years). All age groups were similarly represented except those aged 25–29 (underrepresented by about 45 %) and those aged 40–44 (overrepresented by about 50 %). Almost all participants (98.0 %) were native Italian speakers. A large majority (92.1 %) were living with a partner and two thirds (67.9 %) were employed.

### Headache prevalence

Of the 487 in the primary sample, 406 (83.4 %) reported any headache at some time in their lives. There was a gender-related difference: females 91.2 % (95 % CI: 87.7–94.3), males 72.4 % (95 % CI: 65.8–78.2; *p* < 0.0001); therefore the gender-adjusted lifetime prevalence of any headache was 82.5 %. There was fluctuating variation with age, in the range 86–97 %, between 18 and 49 years; reported prevalence declined then, presumably as a factor of recall, to 79.3 % in those aged 50–54, 69.0 % in those aged 55–59 and 64.4 % in those >60 years.

Of the 487, 373 (76.6 %) reported headache also in the last year, again with a gender difference: females 87.7 % (95 % CI: 84.2–91.8), males 61.1 % (95 % CI: 54.3–67.7; *p* < 0.0001). The gender-adjusted 1-year prevalence of any headache was 74.2 %. Reported 1-year prevalence was clearly age-related, rising to a peak of 96.7 % in the age range 25–29 years, flattening at 86–89 % between 30 and 44 years, then declining steadily, and more rapidly, after age 54 (Table 1).

**Table 1:** Reported 1-year prevalence of any headache in participating sample by age

Age (years)	Proportion reporting headache in thelast year	
	n	% (95 % CI)
18–25	41	78.8 (67.9–90.1)
25–29	29	96.7 (90.9–100)
30–34	41	89.1 (80.0–98.0)
35–39	44	86.3 (76.5–95.5)
40–44	71	88.8 (82.1–95.9)
45–49	42	79.2 (68.0–90.0)
50–54	41	70.7 (59.3–82.7)
55–59	35	60.3 (47.4–72.6)
60–65	29	49.2 (36.2–61.8)

### Type of headache

There were 144 participants (29.6 %) algorithmically diagnosed with definite migraine and another 77 (15.8 %) with probable migraine. The reported 1-year prevalence of all-migraine was 45.5 % (95 % CI: 38.9–52.1 %). This was the most prevalent headache type. Migraine was more prevalent among females (54.6 %) than among males (32.5 %; *p* < 0.0001). The gender-adjusted prevalence of migraine in the participating sample was therefore 42.9 %.

There were 119 participants (24.5 %) with definite TTH and 20 (4.1 %) with probable TTH. The estimated 1-year prevalence of all-TTH was 28.6 % (95 % CI: 21.1–36.1 %). No gender-related difference was observed in the prevalence of TTH (females 28.9 %; males 28.1 %).

Headache on ≥15 days/month was reported by 34 participants (7.0 % [95 % CI: 5.0–9.6 %]). This was very strongly gender-related, since 30 were females; thus the prevalence (based on 1 month’s reporting) of headache on ≥15 days/month was 10.6 % (95 % CI: 7.5–14.7 %) in females and 2.0 % (95 % CI: 0.6–5.1 %) in males (*p* = 0.0002). Among this group were 10 (2.1 % [95 % CI: 1.0–3.8 %]) with pMOH, all of whom were female (*p* = 0.0064).

There were only four cases (0.8 %) of unclassified headache.

### Non-participant sample

A total of 202 questionnaires were completed by non-participants (29.7 % male, 70.3 % female). Of these, 84.7 % reported headache at some time in their lives (not different from the main sample), but only 38.1 % (95 % CI: 31.7–45.0 %) had experienced headache in the past year, far fewer than (in fact half) the 76.6 % (95 % CI: 72.6–80.1 %) of the main sample (*p* < 0.0001). Based on the screening instrument [30], the prevalence of migraine (deemed to be all probable) was 19.8 % (95 % CI: 14.9–25.9 %), less than half that in the main sample (*p* < 0.0001). The prevalence of all causes of headache on ≥15 days/month was 3.5 % and of other headaches 9.6 %.

### Impact of headache

#### Symptom burden

A majority (56.4 %) of those who reported headache in the last year also reported ≥4 episodes of headache in the preceding month (in effect, one or more headache episode per week). Mean days of headache in the sample was 4.8 ± 5.4. On the other hand, one fifth (20.3 %) reported 0–1 attacks. There were 60 (12.3 %) reporting headache on ≥10 days in the last month, including the 34 with headache on ≥15 days.

Since 76.6 % of the sample reported headache with a mean frequency of 4.8 days/month, the probability per person in the sample of having headache on any particular day was 0.12 (0.766*[4.8/30]). Among the sample of 487, 60 would therefore be expected to have headache on any day. One of our questions was “Did you have headache yesterday?”, to which 63 responded positively.

#### Productive time loss

The HALT questionnaire, incorporated into our enquiry, captures headache-attributed lost time from paid work, household work and social life [28]. The 373 participants with headache in our sample, as a group, lost 857 days from paid work in the preceding 3 months, a mean of 2.3 per person, extrapolating to 9.2 days/year or 4.0 % of total working time (assuming 46 weeks worked per year). There was evidence of a highly-disabled small minority, with 20 people (4.1 % of the total sample) reportedly losing >10 days and eight of these (1.6 %) losing >30 days. From household work, 890 days (2.4 per person) were lost, and there were 461 lost social occasions (1.2 per person).

Time losses at individual level were positively correlated with frequency of attacks. For this reason they were greatest in those with headache on ≥15 days/month. They were otherwise dependent on diagnosis, being greater in migraine than in TTH.

#### Impact on professional life

We asked a range of questions pertinent to career and professional prospects, beginning with “Do you feel that your headaches have interfered with your education?” Only 27 (7.2 % [95 % CI: 4.9–10.4 %] of those reporting headache) replied “yes”; specifically, 24 believed they had done less well and three had given up early. Of the 27, most (22) had migraine (10.0 % [95 % CI: 6.6–14.7 %] of those with migraine) but three (30.0 % [95 % CI: 10.3–60.8 %]) had pMOH. Almost the same numbers believed their headaches had hindered their professional careers: in fact, predictably, the same 27 people replied “yes” to this question also, but so did three more with pMOH, to make an overall total of 30 (8.0 % [95 % CI: 5.7–11.3 %]) including six (60.0 % [95 % CI: 31.2–83.3 %]) of those with pMOH. Of these 30, 20 (5.4 % [95 % CI: 3.5–8.2 %]) believed they had done less well, five (1.3 % [95 % CI: 0.5–3.2 %]) had taken an easier job and seven (1.9 % [95 % CI: 0.8–3.9 %]) had required long-term sick-leave (multiple responses were possible to this question). No participant reported early retirement because of headache.

A smaller number (12 [3.2 %; 95 % CI: 1.8–5.6 %]) believed their headaches had limited their incomes, most (9) of whom had migraine while two had pMOH.

Related to employment and work prospects was the question “Do you feel your headaches are understood by your employer and work colleagues?” In all, 50 participants (13.4 % [95 % CI: 10.3–17.3 %] of those with headache) felt not, including not very dissimilar proportions with migraine (13.6 % [95 % CI: 9.0–18.0 %]) and TTH (10.8 % [95 % CI: 5.6–16.0 %]) and three (30.0 % [95 % CI: 10.3–60.8 %] with pMOH.

#### Impact on family life

One way in which headaches interfered with family life was by causing social activities to be missed (reported above). We also enquired into negative effects upon family relationships, childcare, family planning and love life, but our sample was not large enough to support a worthwhile analysis of these.

We asked: “Do you feel your headaches are understood by your family?” Only 31 participants (8.3 % [95 % CI: 5.9–11.6 %] of those with headache) responded “no”, spread through the diagnostic groups, and we did not analyse these further.

#### Impact on quality of life

Most (340: 69.8 %) of the 487 participants considered their quality of life good or very good, and there was little difference between those with no headache (70.1 %), migraine (69.7 %) or TTH (72.7 %). Of the 10 participants with pMOH, however, only two (20.0 % [95 % CI: 4.6–52.1 %]; *P* = 0.0019) reported good or very good quality of life.

We asked “How satisfied are you with your ability to perform your daily living activities?” [29]. Of the total sample, 70.4 % were satisfied or very satisfied, as were very similar proportions with migraine (70.1 % [95 % CI: 63.8–75.8 %]) or TTH (74.8 % [95 % CI: 67.0–81.3 %]). Among those with pMOH, satisfaction was much lower (10.0 % [95 % CI: 0.01–42.6 %]).

### Use of health care

Despite the high prevalence of headache in our sample, only 16.6 % of participants reporting headache had received a diagnosis from a doctor. Only 36 (9.7 % of those reporting headache) had consulted a primary-care physician in the last year; 18 (4.8 %) had reached a headache specialist; three (0.8 %) had been seen in a hospital emergency room.

The great majority (84.0 %) of those with headache had used symptomatic drugs for headache attacks in the last 30 days, whereas only 2.4 % were taking preventative medication. The use of preventative medication was higher among participants with headache on ≥15 days/month, but numbers were too small to analyse reliably.

## Discussion

We need first to consider the limitations of this study, because there were several. The Eurolight Project itself “was a very large and organizationally complex study, involving multiple collaborating partners (academic and lay) in ten countries [*making*] pragmatic methodological compromises in order to complete it” [18]. It had considerable strengths: a questionnaire already used and validated by LTB in many different countries, cultures and translations (the HARDSHIP questionnaire [17]); neutral screening questions expected to lead to better ascertainment than questions incorporating degrees of frequency or severity [7]; diagnoses made according to a standard algorithm also developed and widely used by LTB [17]. Nevertheless, Eurolight could not undertake diagnostic validation in each of the translations used, so diagnostic accuracy was not directly assessed. This was one limitation here. The modified cluster-sampling approach of Eurolight, using different sampling methods in different countries, was seen as a strength of the main project [18], since it was not a primary purpose of Eurolight to generate representative data at national level. For this, its sampling methods were less suited, so this was a second limitation here. The third key limitation was the non-participation rate of 85.7 %.

With regard to diagnosis, we believe the instrument was reliable in view of its successful use elsewhere [17], and we draw attention as evidence of this to the expected gender differential in migraine but not TTH. Nonetheless, we are circumspect about making comparisons between migraine and TTH, which were not the main purpose of this report. Headache on ≥15 days/month was diagnosed (as a group of disorders) simply on the basis of reported frequency, and, among these, pMOH on the basis additionally of reported medication use. We see very clear differentials in impact between the episodic headaches and pMOH. The key messages of this report relate to headache disorders.

For the Eurolight project we sampled from one area of Italy: Pavia province in Lombardy region, Northern Italy, an area of industry, high population density and (relative to Italy as a whole) high gross domestic product (GDP). It is not representative of the country. Nevertheless, sampling, carefully stratified, was from the general population across the area, urban and rural; unless there are cultural, environmental or genetic factors varying across Italy that greatly influence the prevalence or expression of headache disorders, the findings from this area should at least be indicative for Italy as a whole. The sample, mostly of employed people living with partners, was good for investigating the impact of headache on the different aspects of life.

Much more troublesome is the high non-participation rate — not, of course, unusual in a postal survey. There was clear evidence of interest-bias, as might be expected; the non-participant survey recorded prevalences that were less than half those reported by the primary responders. A special reason for interest bias might be that, in Pavia, we have had a very active headache centre for over 30 years; many who participated may have been followed by the centre in the past, and responded because they were sensitive to the issue. However, it would not be correct to assume that this smaller sample, responding to a much shorter questionnaire, provided more reliable data.

With these limitations of our own study in mind, we review other available data. In Italy, as we noted, the epidemiology of headache disorders is not well established, and data on impact are very few. One study only has been performed on a sample representative of the general population [31], but this predated and therefore could not use accepted diagnostic criteria. It involved the small independent Republic of San Marino, and administered a questionnaire face-to-face to a random sample of 1500 subjects over 7 years of age summoned by mail. Of the 76.3 % who agreed to be interviewed, 528 (46.1 %) reported headache during the previous year. One-year prevalence of migraine was estimated at 9.3 % in males and 18 % in females. More recent but less population-based was the sample of 71,588 patients interviewed by 902 general practitioners (GPs) [32]. All patients who visited the GPs for any reason during five consecutive days of two different weeks were asked whether they suffered from headache; if they did, the GP completed a diagnostic questionnaire based on ICHD-I [23]. The estimated prevalence of migraine in this sample was 11.6 % (5.0 % in males and 15.8 % in females).

Other epidemiological studies in Italy have focused on selected groups. Prencipe et al [19], surveyed 1147 people aged ≥65 years in three rural villages in central Italy, 72.6 % of whom completed the protocol, including clinical evaluation by a neurologist and diagnosis according to ICHD-I [23] (with minor modifications in order to classify patients with headache on ≥15 days/month, then referred to as “chronic daily headache” [CDH]). Estimated 1-year prevalence of migraine was 11.0 %, of TTH 44.5 % and of CDH 4.4 % (with one third of these being pMOH). The similar study of 1031 residents aged ≥65 years of a rural village in southern Italy found that 225 (21.8 %) suffered from recurrent headaches (defined as ≥3 attacks within the past 12 months) [20]. Estimated 1-year prevalence of migraine was 4.6 %, of TTH 16 % and of pMOH 1.3 %. Both migraine and TTH were significantly more prevalent among females than males; only migraine prevalence significantly decreased with increasing age. Raieli et al surveyed 1445 students aged 11–14 years, diagnosing migraine by questionnaire and neurological examination and using the criteria of ICHD-I [23]. Prevalence of migraine without aura was 2.35 %, of migraine with aura 0.62 %.

More recently, Ferrante and colleagues [24] collaborated with GPs to survey the prevalence and clinical features of primary headaches in an adult population of the Emilia-Romagna region in Northern Italy. They evaluated 904 subjects using face-to-face interviews by a specialist of the Headache Centre of Parma. The 1-year adjusted prevalence of definite migraine (including chronic migraine) was 24.7 % and of probable migraine 5.1 %.

The emerging picture is far from clear. Ferrante’s study indicates a prevalence of all-migraine of 29.8 %, about one third lower than our gender-adjusted 42.9 % but considerably higher than the 19.8 % of our non-participant sample. While the mean global prevalence of migraine was estimated at 14.7 % in GBD2010 [11], several other LTB studies estimating the 1-year prevalence of migraine have found values in the range 20–26 %: in Russia 20.8 % [10]; in India 25.6 % [9]; in Zambia 22.9 % [33]; in Pakistan 22.9 % [34]. Ferrante’s finding is not unreasonable in the light of these, and we would say it is closer to the truth than ours, since we must recognise in ours the effect of interest bias. Eurolight was more focused on impact than prevalence, and it is insights into impact that this report is able to add.

As regards TTH, Ferrante et al [25] reported a crude prevalence of 19.4 %, similar to the 21.2 % reported by Vukovic in nearby Croatia [35], while our reported prevalence of 28.6 % is closer to the 30.9 % observed by Ayzenberg et al in Russia [10]. Our finding (as, indeed, that of Ayzenberg et al, who used a similar questionnaire) was subject to systematic underestimation since we diagnosed only one headache type (the most bothersome) in any participant with more than one. The ample variability in TTH prevalence that can be found in the literature (from 5.1 to 74.0 % [36–38], is probably dependent on a multiplicity of reasons, but more on variable methodological approaches and cultural differences in reporting the often mild and infrequent headache of TTH than true differences between populations due to geographical, demographic, racial, economic and psychosocial factors. This consideration underscores the need of national data for the correct implementation of adequate health care policies.

We saw that more than half (56.4 %) of those reporting headache in the last year were experiencing one or more headache episodes per week. One participant in eight (12.3 %) reported headache on ≥10 days in the last month. The frequencies reported generated an expectation that 60 of the sample of 487 would have headache on any one day. In fact, 63 reported headache yesterday, strongly supporting the responses based on recall (and validating the enquiry).

More than two thirds of our sample were employed. Each person with headache lost, on average, 2.3 days from paid work in the preceding 3 months, extrapolating to 9.2 days/year or 4.0 % of total working time. Spread between the entire sample of 487, this would represent a huge 3.1 % loss to GDP. Much of this was attributable to the highly-disabled small minority – the 20 people (4.1 % of the sample) losing >10 days and, particularly, the eight of these (1.6 %) losing >30 days. Even so, we suspect this finding was also influenced by interest bias; since losses at individual level were greater from migraine than from TTH, we suggest a conservative downrating of the estimate by one third, in line with the prevalence estimate of Ferrante et al [24] rather than our own. The revised loss to GDP of 2.0 % still exceeds estimates made by LTB for Russia (1.75 % [10]), China (1.9 % [8]) and Zambia (1.9 %) [33]). It should be noted that losses from household work were equally great, and these are also economically important.

People with difficulties at work were not helped by employers or colleagues who offered no understanding (in 50 reported cases), perhaps unsurprisingly when they bore the burden of the headache sufferer’s absenteeism. At home, 31 felt a lack of understanding. Nonetheless, most people with headache were not aware of negative consequences on education, career and professional prospects, family life or quality of life, although in each of these domains there was a disaffected minority. The consequences for the 27 (most with migraine) who believed they had done less well at school, or given up early, were potentially cumulative through a lifetime. So were they for the 30 who believed their headaches had hindered their careers, and the 12 who believed their headaches had limited their incomes. Again, most had migraine. Still the great majority of people with headache – at least those with episodic headache – seemed to get on with their lives. The two quality-of-life questions showed no differences between those with no headache, those with migraine and those with TTH, although those with pMOH scored well below all others on both questions.

Despite that 76.6 % of our sample reported headache within the last year, only 16.6 % of these had a doctor’s diagnosis and, in the last year, only 9.7 % had consulted a primary-care physician and 4.8 % a headache specialist. Here, interest bias is likely to mean that these numbers are higher than the norm (*ie*, a *better* picture than reality). There are many reasons for consultation-failure, including patient inaction. Torelli et al [39] assessed disease perception among a sample of 904 subjects; of 387 (123 males, 264 females) who reported headache in the past year, only one third perceived themselves as “headache sufferers”, this perception being dependent on headache frequency in females only. But, whatever its cause, the end-result of health-care failure, so evident in our study with only 2.4 % taking preventative medication despite that more than half the sample reported ≥4 headache days/month, is unrelieved burden, including symptom burden, disability, productive time losses and the inordinate losses to GDP.

## Conclusions

Eurolight is not, generally, a source of reliable epidemiological data [18], a point we emphasise. The data from Italy were population-based, but from a selected area of the industrial north; furthermore, there was low participation. Eurolight’s strength was its detailed enquiry into impact. Taking other prevalence data into account, a fair picture can be painted of headache in Italy. As in other countries, headache disorders are highly prevalent and burdensome. In Italy, the high impact on individuals is matched by high economic burden; both are unmitigated by health-care failure. There is a highly-disabled small minority. Health policy in Italy must take note of these findings, of suggestions that Italy fares worse than other countries, and of the World Health Organization’s advice that effective treatment of headache is desirable not only for its health benefits but also because it is likely to be cost-saving [40].
